# Supplementary material for: Diffusion interface layer controlling the acceptor phase of bilayer near-infrared polymer phototransistors with ultrahigh photosensitivity
Source: Nat Commun. 2022 Mar 11;13:1332. doi: 10.1038/s41467-022-28922-4 (PMC8917130; doi:10.1038/s41467-022-28922-4)
Supplement: Supplementary file 1 — Supplementary Information [file 41467_2022_28922_MOESM1_ESM.pdf]

Supplementary Information

Diffusion Interface Layer Controlling the Acceptor Phase of Bilayer Near-Infrared Polymer Phototransistors with Ultrahigh Photosensitivity

Tao Han[*,1], Zejiang Wang[1], Ning Shen[1], Zewen Zhou[1], Xuehua Hou[2], Shufang Ding[1], Chunzhi Jiang[1], Xiaoyi Huang[1], Xiaofeng Zhang[*,3], Linlin Liu[2]

[1]Hunan Provincial Key Laboratory of Xiangnan Rare-Precious Metals Compounds Research and Application, School of Physics and Electronic Electrical Engineering, Xiangnan University, Chenzhou 423000, P. R. China.

[2]Institute of Polymer Optoelectronic Materials and Devices, State Key Laboratory of Luminescent Materials and Devices, South China University of Technology, Guangzhou 510640, P. R. China.

[3]National Engineering Laboratory for Modern Materials Surface Engineering Technology & The Key Lab of Guangdong for Modern Surface Engineering Technology, Guangdong Institute of New Materials, Guangzhou 510650, P. R. China.

[*] Corresponding authors:

Tao Han (than@xnu.edu.cn), Xiaofeng Zhang (zxf200808@126.com).

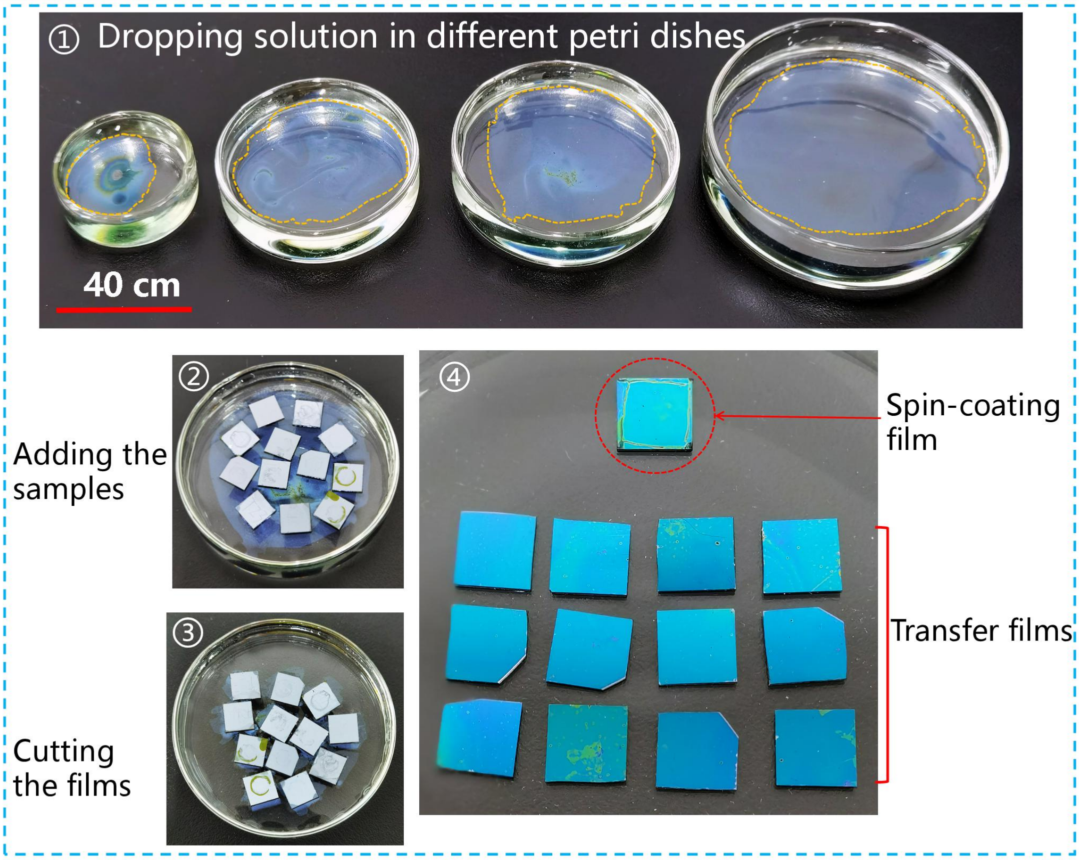

Supplementary Fig. 1 Practical fabrication of films via floating film transfer method (FTM). Here, the areas of the devices in the ②-④ are 1 × 1 cm^2.

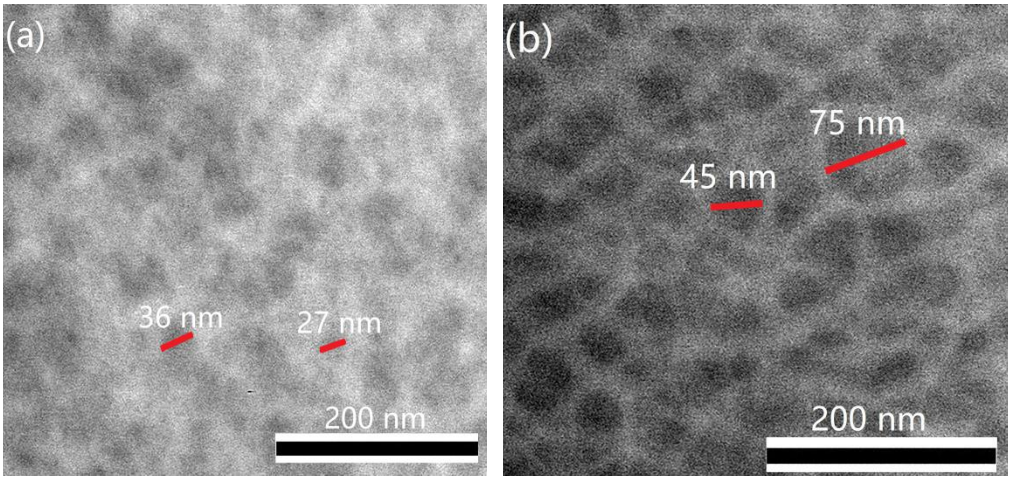

Supplementary Fig. 2 Locally enlarged TEM images of the active layer in the Fig. 2a. **a** Spin-coated PDPP3T:$PC_{61}BM$. **b** Transfered PDPP3T:$PC_{61}BM$.

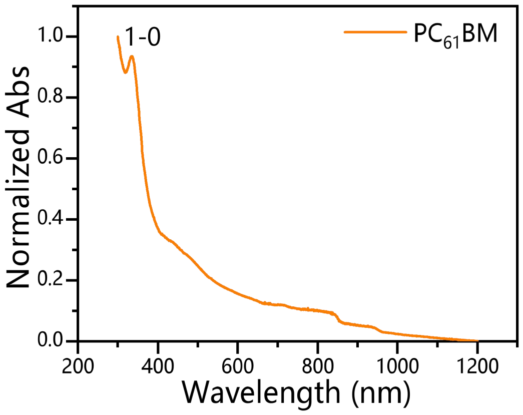

Supplementary Fig. 3 The UV–vis absorption spectra of the $PC_{61}BM$ film. Here, 1-0 absorption peak at 334 nm.

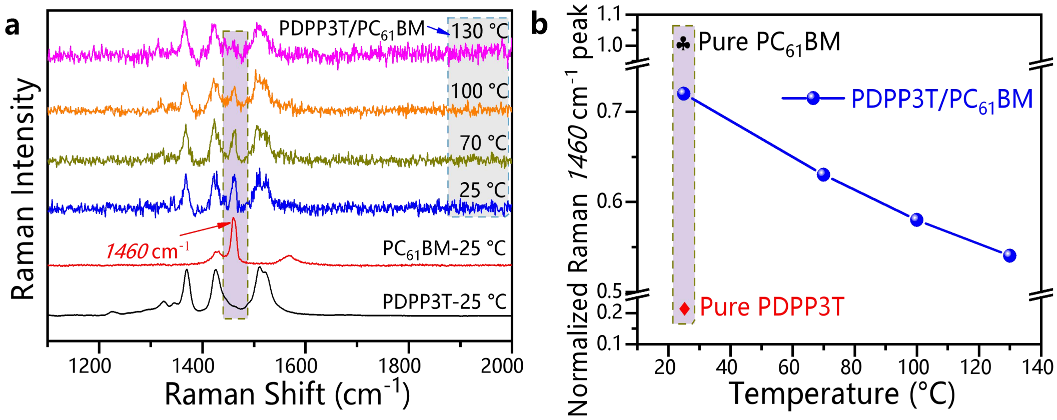

Supplementary Fig. 4 The in situ Raman spectra. a In situ Raman spectra of the PDPP3T/$PC_{61}BM$ bilayers with solvent vapor annealing (SVA) treatment at different temperatures. **b** Dependence of normalized $PC_{61}BM$ Raman peak in PDPP3T/$PC_{61}BM$ layer on annealing temperature (data comes from Supplementary Fig. 4a, ♣ represents the $PC_{61}BM$ film, ♦ represents the PDPP3T film). Here, the PDPP3T-25 °C and $PC_{61}BM$-25 °C represent the Raman tested of PDPP3T and $PC_{61}BM$ film at 25 °C in Supplementary Fig. 4a.

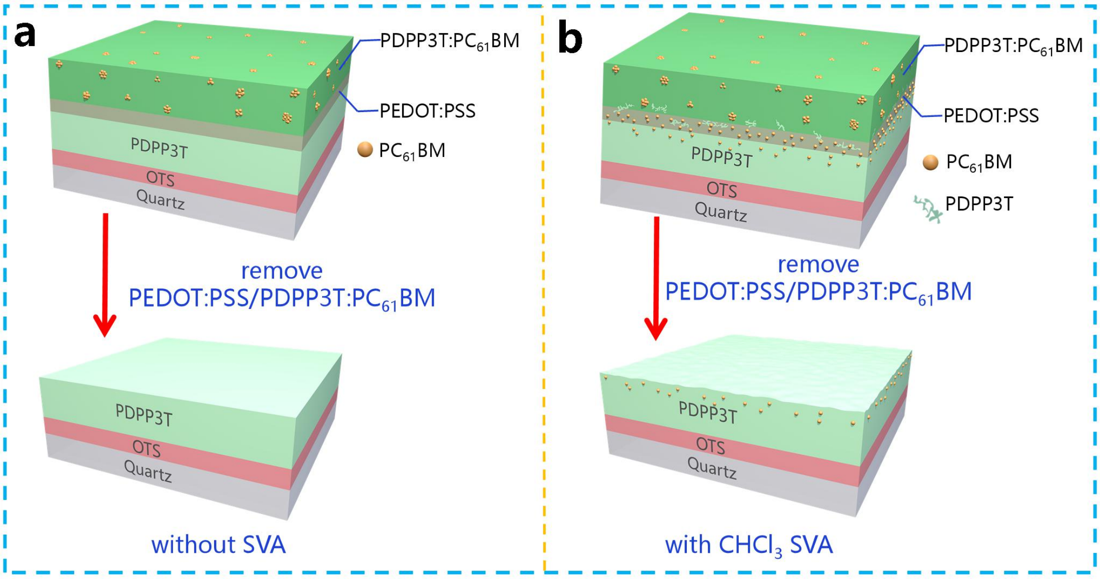

Supplementary Fig. 5 Schematic diagram of PEDOT:PSS/PDPP3T:$PC_{61}BM$ layer is removed from quratz/ OTS/PDPP3T/PEDOT:PSS/PDPP3T:$PC_{61}BM$ structure. **a** The films without SVA. **b** The films with SVA.

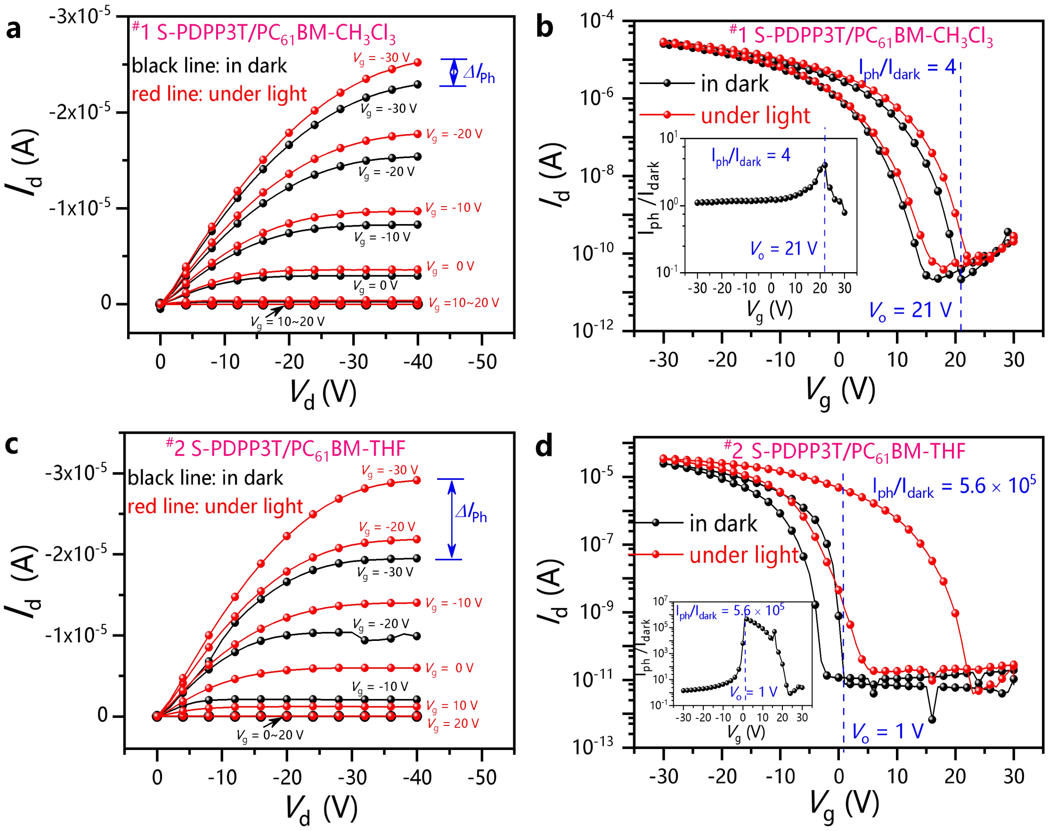

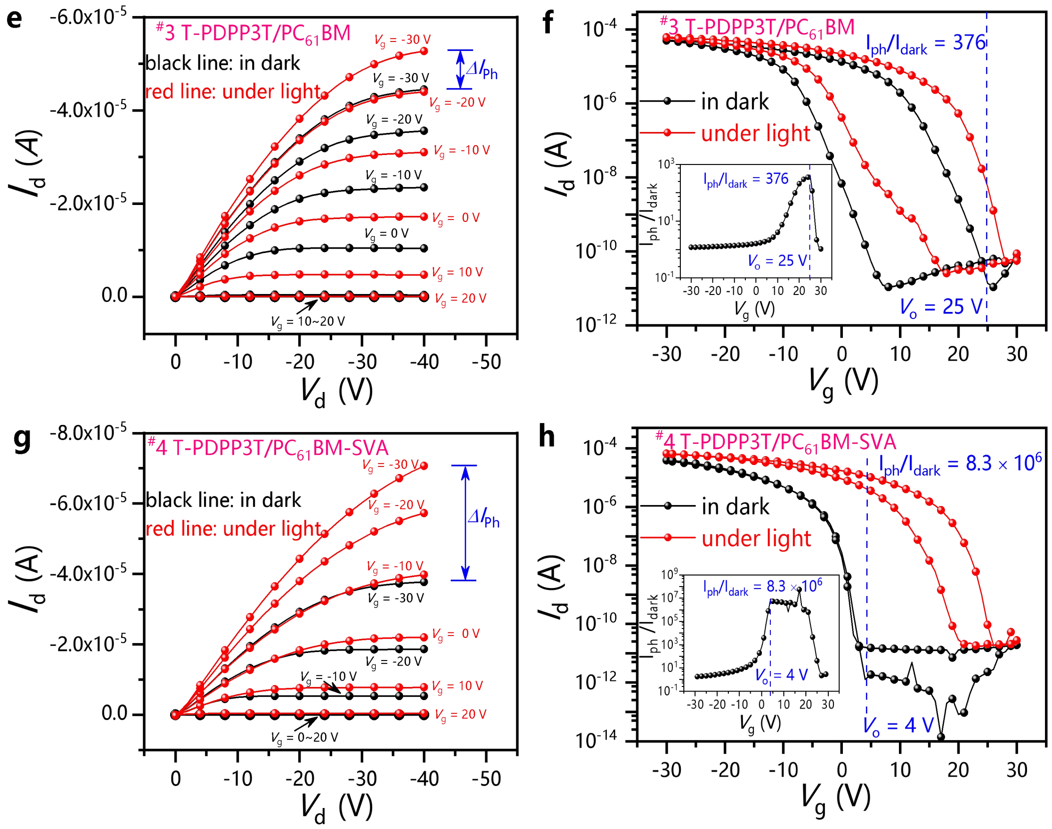

Supplementary Fig. 6 Electrical properties of devices with Si/SiO_2/OTS/PDPP3T/Au/ $PC_{61}BM$ structure via different fabrication methods. Output curve **a** and transfer curve **b** of Deivce #1 S-PDPP3T/$PC_{61}BM$-CH_3Cl_3. Output curve **c** and transfer curve **d** of Deivce #2 S-PDPP3T/$PC_{61}BM$-THF. Output curve **e** and transfer curve **f** of Deivce #3 T-PDPP3T/$PC_{61}BM$. Output curve **g** and transfer curve **h** of Deivce #4 T-PDPP3T/$PC_{61}BM$-SVA. The light intensity is 0.04 mW/cm^2 @ 850 nm. The transfer curve were tested with V_d = -30 V.

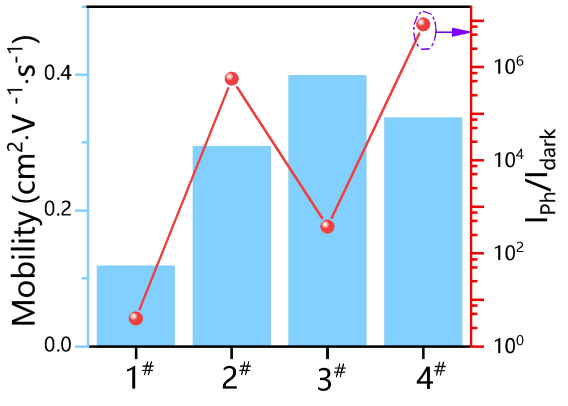

Supplementary Fig. 7 The electrical properties of devices prepared with different methods: hole mobility and I_{ph}/I_{dark}.

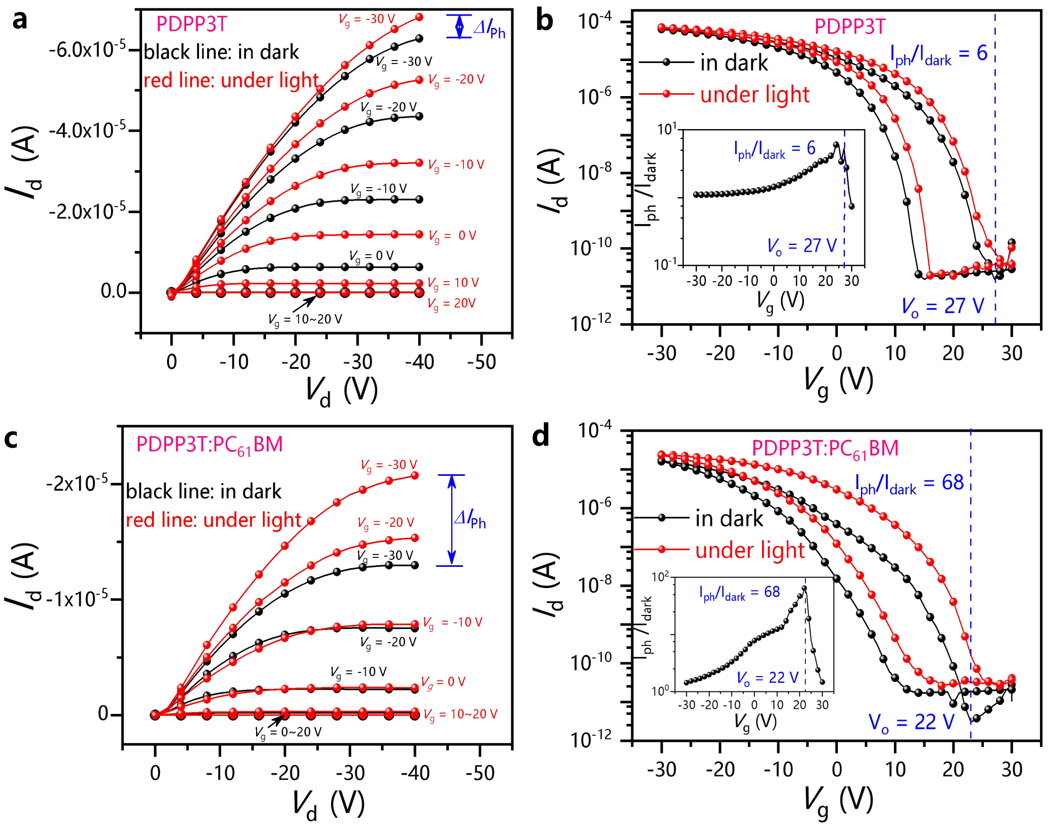

Supplementary Fig. 8 Electrical properties of PDPP3T device and PDPP3T:PC_{61}BM devices. Output curve **a** and transfer curve **b** of PDPP3T device. Output curve **c** and transfer curve **d** of PDPP3T:PC_{61}BM device. The light intensity is 0.04 mW/cm^2 @ 850 nm. The transfer curves of the devices were measured at a constant V_d = -30 V.

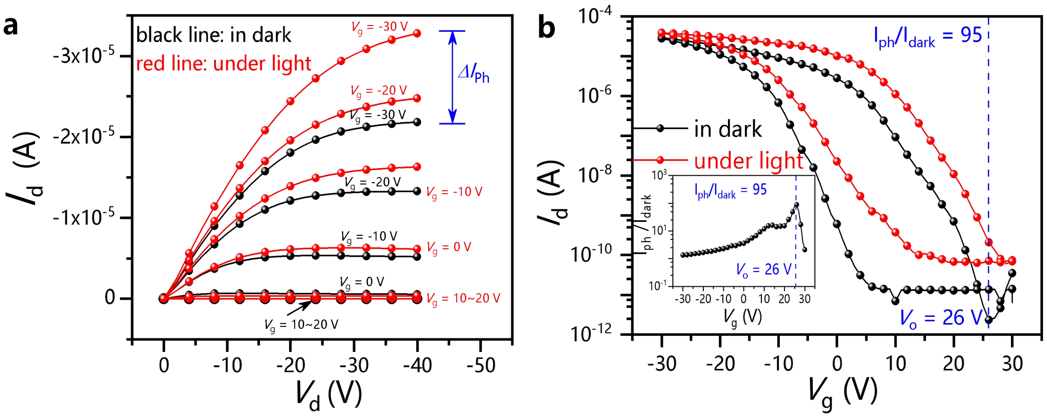

Supplementary Fig. 9 Electrical properties of the device with structure of Si/SiO_2/OTS/Au/PDPP3T:PC_{61}BM. Output curve **a** and transfer curve **b** of the device with structure of Si/SiO_2/OTS/Au/PDPP3T:PC_{61}BM. The light intensity is 0.04 mW/cm^2 @ 850 nm. The transfer curves of the devices were measured at a constant V_d = -30 V.

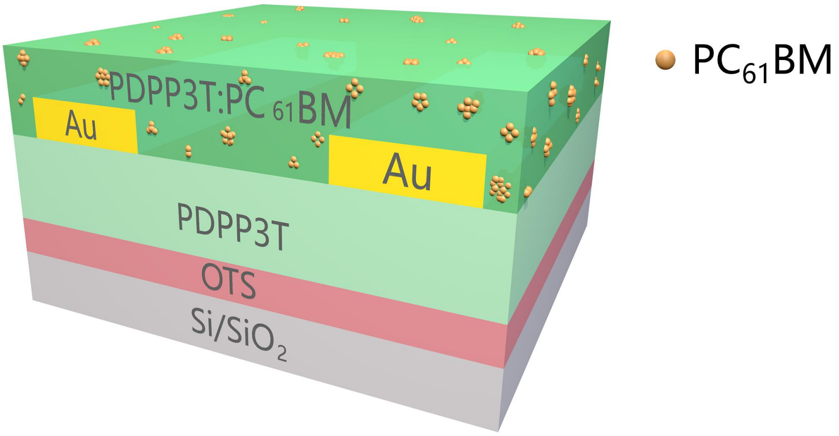

Supplementary Fig. 10 Schematic diagram of Device #5 W/O SVA: Si/SiO_2/OTS/ PDPP3T/Au/PDPP3T:$PC_{61}BM$. Here, the device was prepared by FTM without SVA treatment.

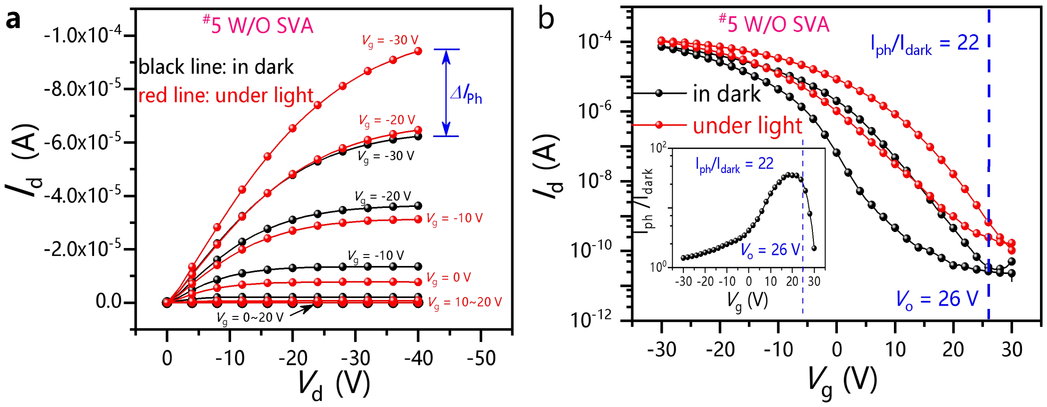

Supplementary Fig. 11 Electrical properties of the Device #5 W/O SVA. Output curve **a** and transfer curve **b** of Device #5 W/O SVA. The light intensity is 0.04 mW/cm^2 @ 850 nm. The transfer curves of the devices were measured at a constant V_d = -30 V.

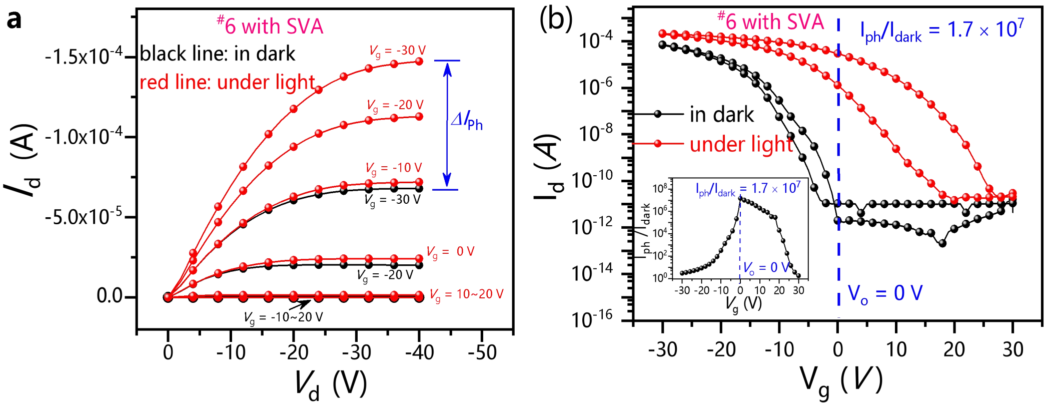

Supplementary Fig. 12 Electrical properties of the Device #6 with SVA. Output curve **a** and transfer curve **b** of Device #6 with SVA. The light intensity is 0.04 mW/cm^2 @ 850 nm, and the SVA temperature is 100 °C for 20 minutes. The transfer curves of the devices were measured at a constant V_d = -30 V.

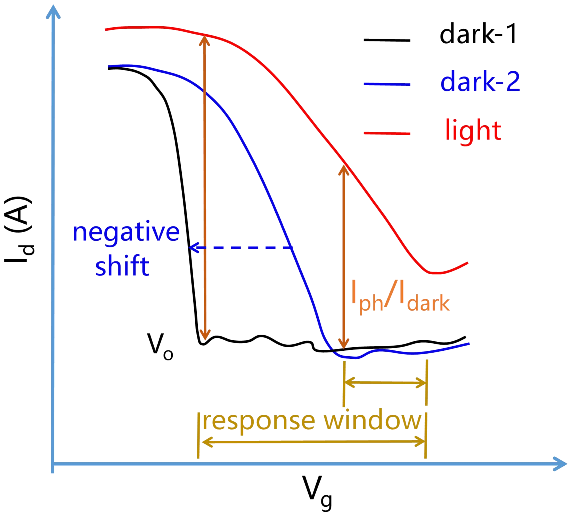

Supplementary Fig. 13 The effect of threshold voltage drift on the value of I_{ph}/I_{dark} and response window.

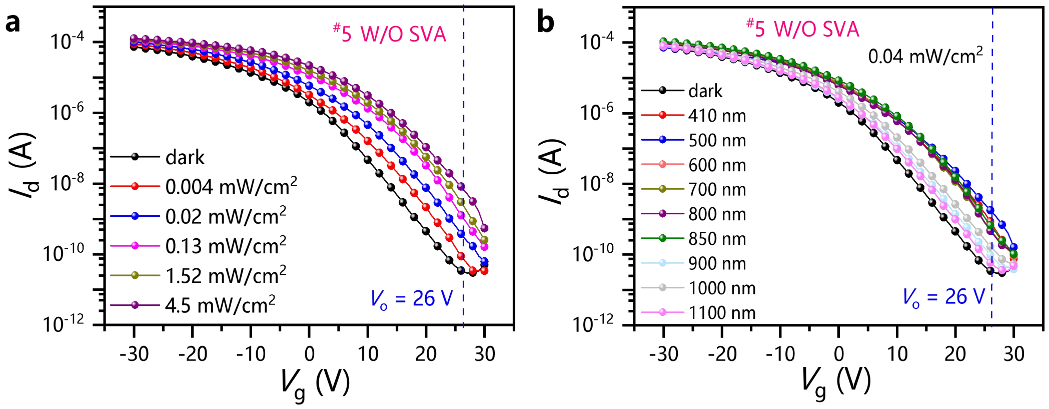

Supplementary Fig. 14 The transfer curve of Device #5 W/O SVA. a Different light intensity under 850 nm. **b** Different light wavelength at 0.04 mW/cm^2. The transfer curves of the devices were measured at a constant V_d = -30 V.

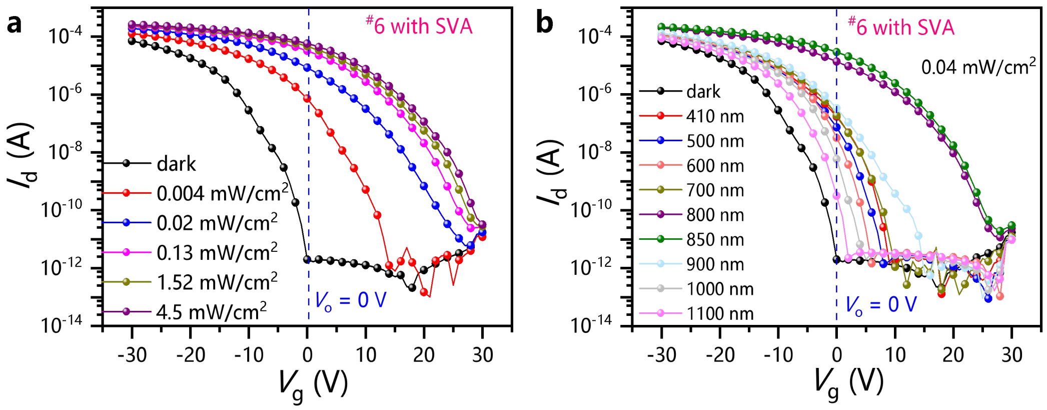

Supplementary Fig. 15 The transfer curve of Device #6 with SVA. a Different light intensity under 850 nm. **b** Different light wavelength at 0.04 mW/cm^2. The SVA temperature is 100 °C for 20 minutes. The transfer curves of the devices were measured at a constant V_d = -30 V.

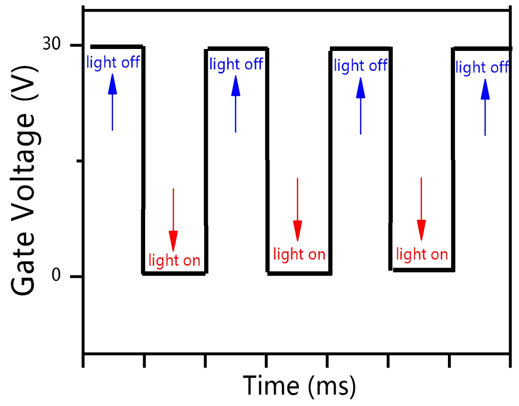

Supplementary Fig. 16 The schematic diagram of gate and light co-control method of OPTs.

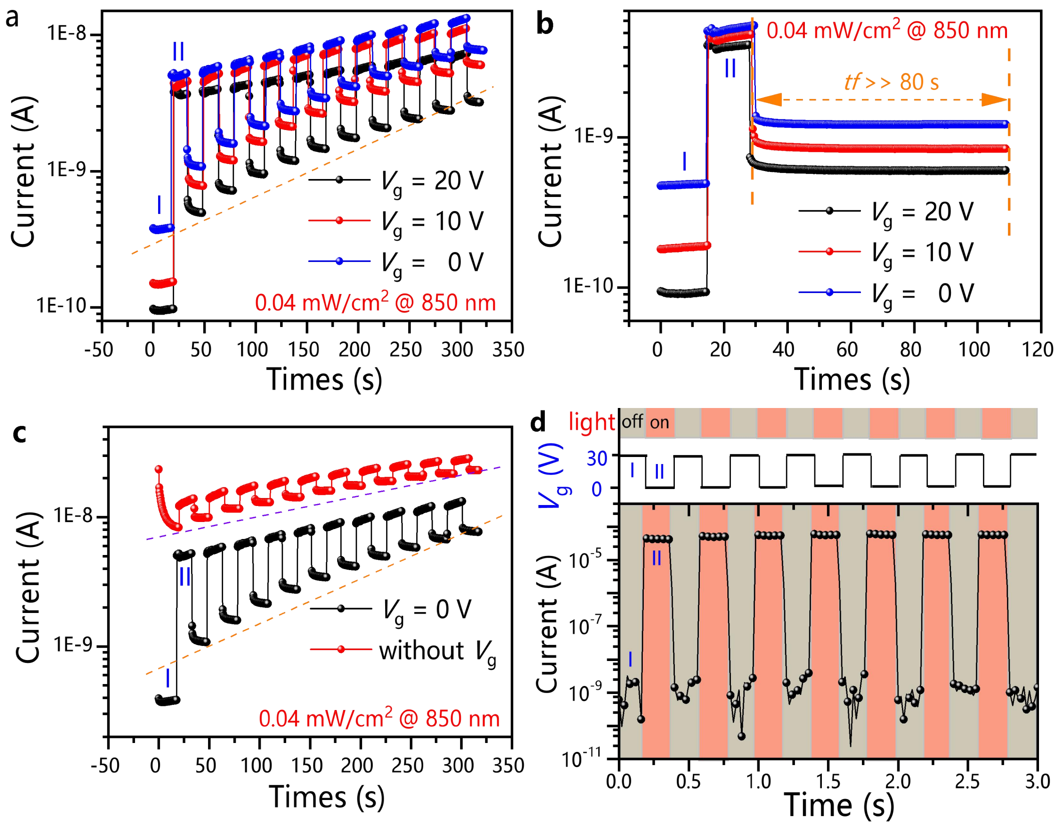

Supplementary Fig. 17 The time-current curves of Device #6 with SVA at a constant source-drain voltage (V_d = -30 V) under light excitation (0.04 mW/cm^2 @ 850 nm). a Multiple switching cycles with different gate voltage (V_g). **b** Single switching cycle with different gate voltage (V_g). **c** With V_g = 0 V (three-terminal device) and without gate voltage (two-terminal device). **d** The gate and light co-control method testing. Ⅰ represents light-off, Ⅱ represents light-on.

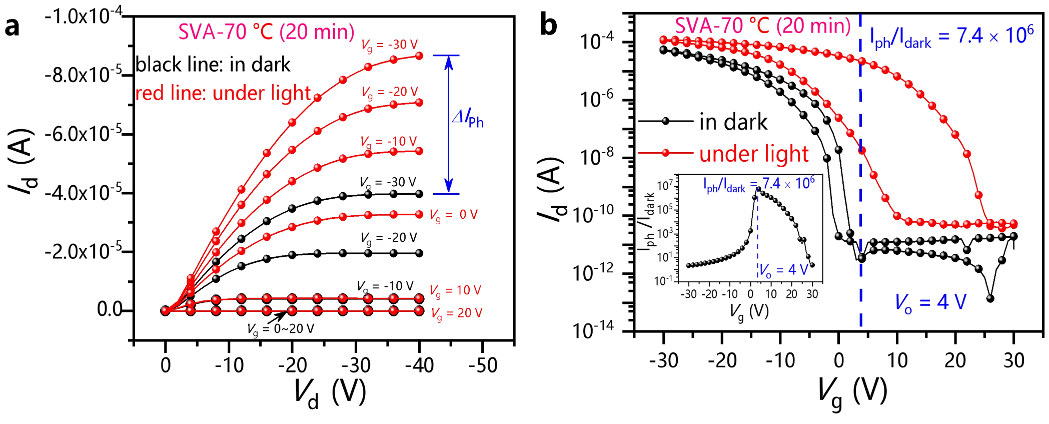

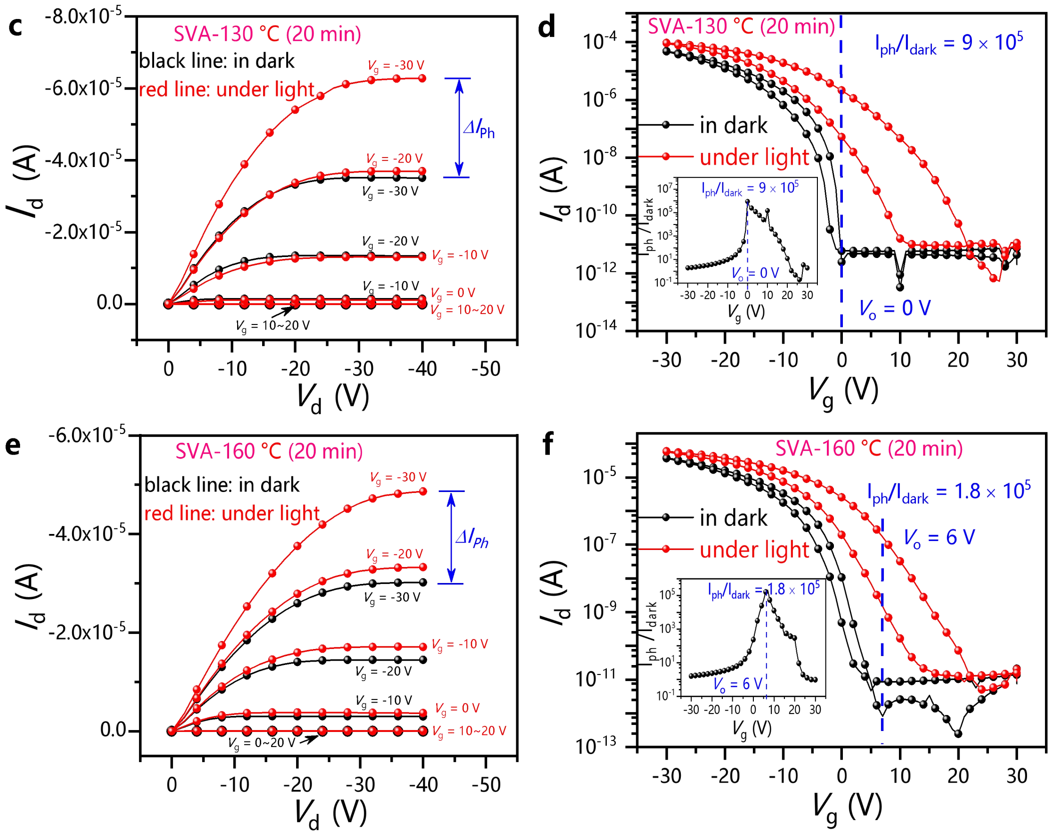

Supplementary Fig. 18 The electrical properties of SVA devices (20 minutes) with different temperatures. Output curve **a** and transfer curve **b** of the device with 70 °C SVA treatment. Output curve **c** and transfer curve **d** of the device with 130 °C SVA treatment. Output curve **e** and transfer curve **f** of the device with 160 °C SVA treatment. The light intensity is 0.04 mW/cm^2 @ 850 nm. The transfer curves of the devices were measured at a constant V_d = -30 V.

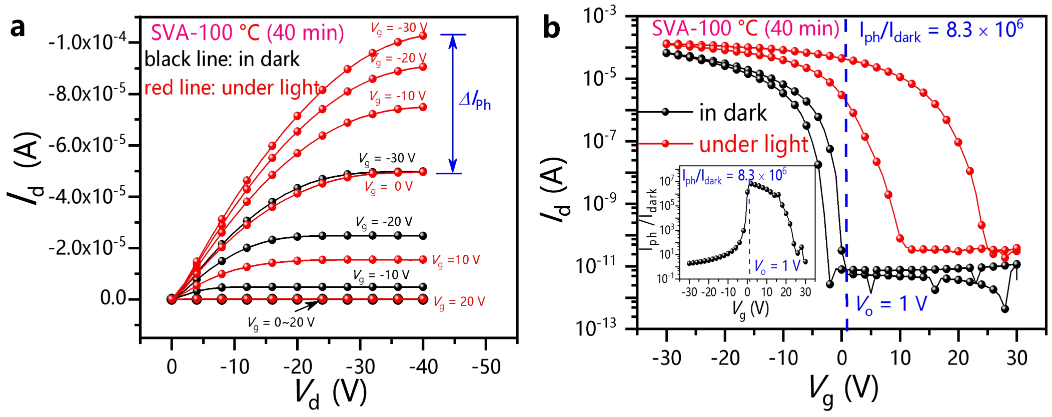

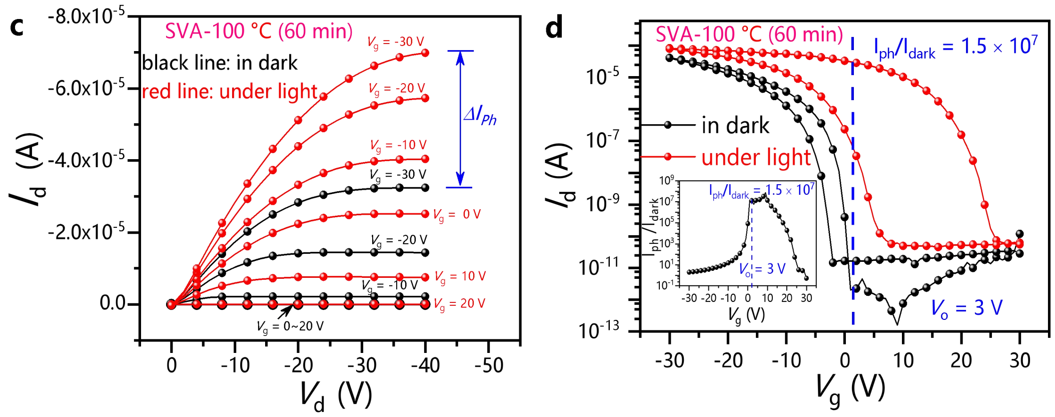

Supplementary Fig. 19 The electrical properties of SVA devices (100 °C) with different time. Output curve **a** and transfer curve **b** of the device with 40 minutes SVA treatment. Output curve **c** and transfer curve **d** of the device with 60 minutes SVA treatment. The light intensity is 0.04 mW/cm^2 @ 850 nm. The transfer curves of the devices were measured at a constant V_d = -30 V.

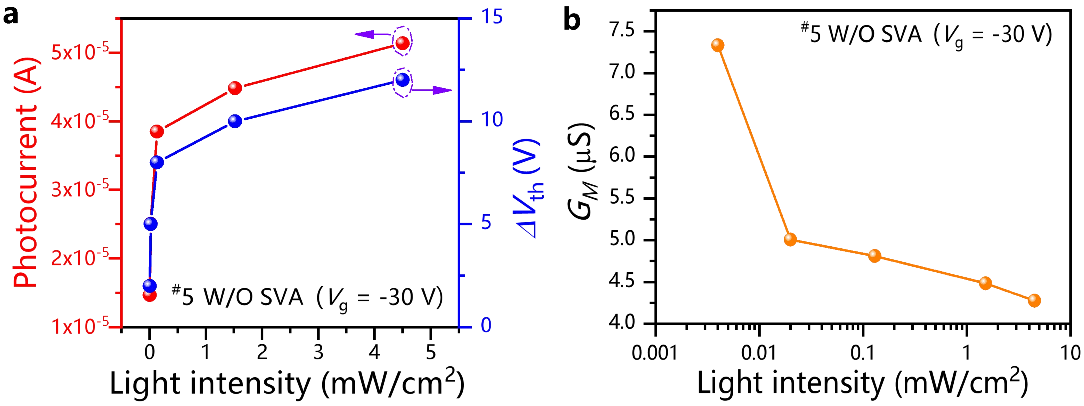

Supplementary Fig. 20 Dependence of photocurrent (ΔI_{ph}), threshold voltage drift (ΔV_{th}) and G_M on light intensity of the Device #5 W/O SVA at V_g = -30 V. a Dependence of ΔI_{ph} and ΔV_{th} on light intensity. **b** Dependence of G_M on light intensity.

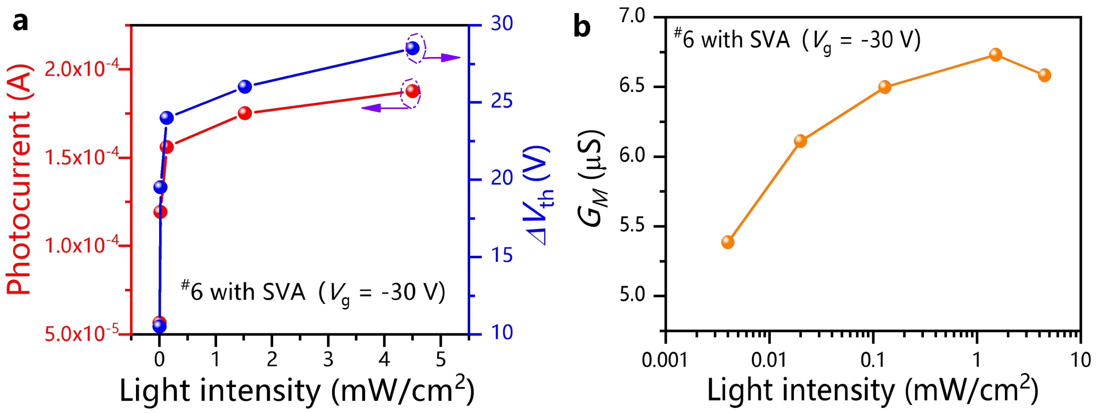

Supplementary Fig. 21 Dependence of photocurrent (ΔI_{ph}), threshold voltage drift (ΔV_{th}) and G_M on light intensity of the Device #6 with SVA at V_g = -30 V. a Dependence of ΔI_{ph} and ΔV_{th} on light intensity. **b** Dependence of G_M on light intensity.

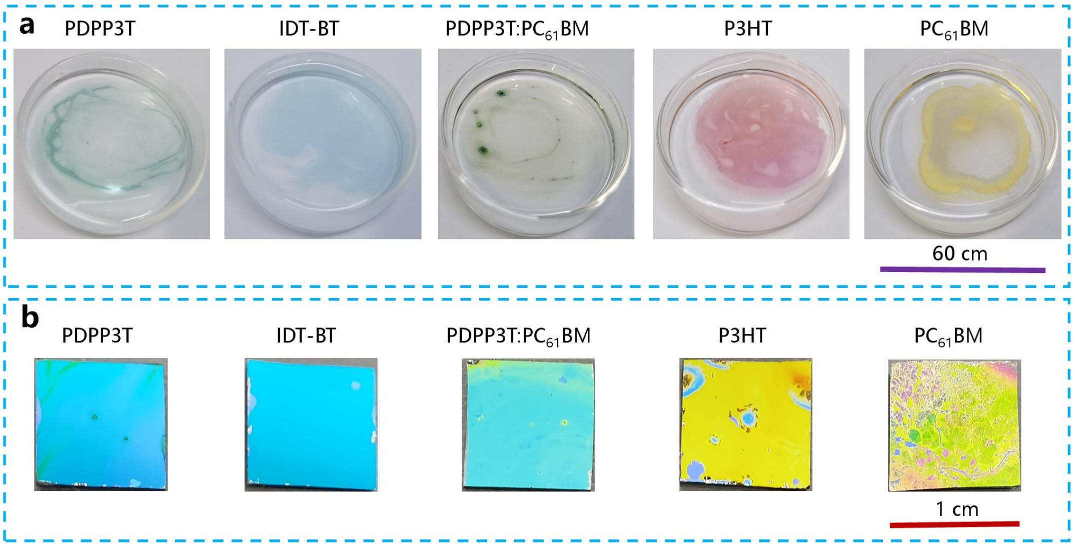

Supplementary Fig. 22 Different solutions expansion and films surface photographs via FTM. a The different solutions expansion on the deionized water surface. **b** The different films surface photographs via FTM.

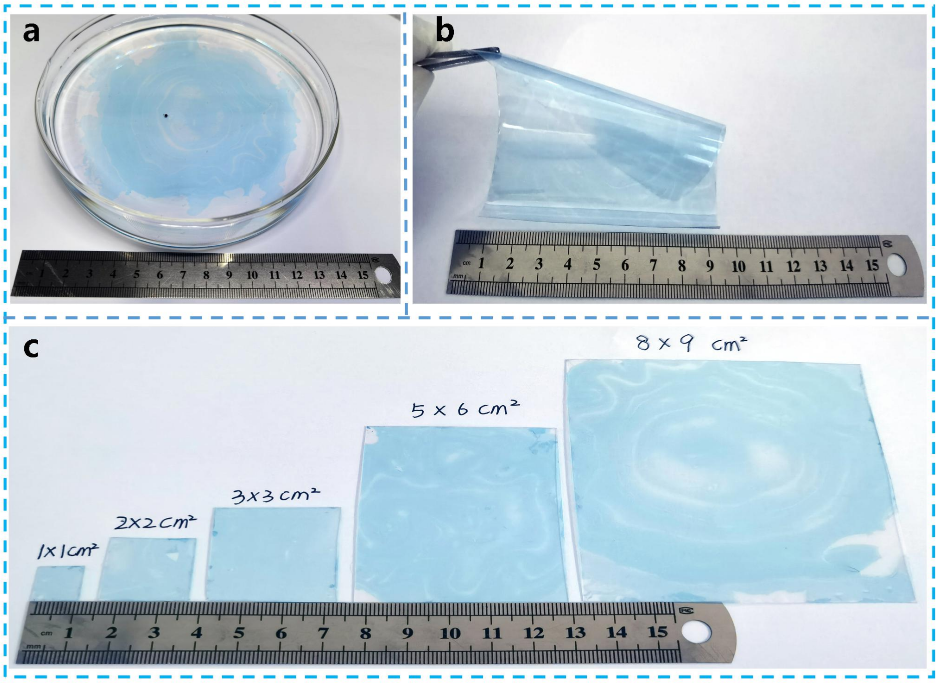

Supplementary Fig. 23 IDT-BT solution expansion and film surface photographs via FTM. a The IDT-BT solution expansion on the deionized water surface. **b** The IDT-BT film photographs via FTM on the flexible substrate. **c** The IDT-BT film photographs with different sizes via FTM.

Supplementary Table 1. Summary of the UV-vis spectra and UPS analysis.

	UV-vis absorption		UPS			
	λ_{onset} (nm)[a]	E_g^{opt} (eV)[b]	E_{Cutoff} (eV)	E_V-E_F (eV)	E_{HOMO} (eV)[c]	E_{LUMO} (eV)[d]
PDPP3T	918	1.35	18.79	2.81	5.24	3.89
$PC_{61}BM$	666	1.86	18.8	3.54	5.96	4.1

[a] the absorption onset wavelength of the film, [b] optical bandgap calculated via λ_{onset}, [c] HOMO levels obtained by the E_{Cutoff} and E_V-E_F values, [d] LUMO levels determined by the E_{HOMO} and E_g^{opt}.

Supplementary Table 2. Device structures with different preparation method.[a]

Device	Dielectric layer	First layer	Electrode	Seceond layer
PDPP3T		PDPP3T (transferred)		None
PDPP3T:$PC_{61}BM$	SiO_2 (300 nm)/OTS	PDPP3T:$PC_{61}BM$ (transferred)	Au	None

[a] The thicknesses of the PDPP3T film and the PDPP3T:$PC_{61}BM$ film via the film transfer method are 70 and 60 nm, respectively.

Supplementary Table 3. Performance parameters of different devices.

Device	ΔI_{ph} (μA)	V_o (V) in the dark	Off-state current (μA)	Mobility ($cm^2 \cdot V^{-1} \cdot s^{-1}$)	I_{ph}/I_{dark}
PDPP3T	5.3	27	2.0×10^{-5}	0.307	6
PDPP3T:$PC_{61}BM$	7.8	22	3.0×10^{-6}	0.136	68

Supplementary Table 4. Device structures with and without SVA treatment.[b]

Device	Dielectric layer	First layer	Electrode	Seceond layer
#5 W/O SVA				PDPP3T:$PC_{61}BM$ (transferred and without SVA)
	SiO_2 (300 nm)/OTS	PDPP3T (transferred)	Au	
#6 with SVA				PDPP3T:$PC_{61}BM$ (transferred

	and with SVA)

[b] The thicknesses of the PDPP3T film and the PDPP3T:$PC_{61}BM$ film via the film transfer method are 70 and 60 nm, respectively.

Supplementary Table 5. Performance parameters of devices with and without SVA treatment.

Light intensity (mW/cm^2)	0.004	0.02	0.13	1.52	4.5
ΔV_{th} (V) - Device #5 W/O SVA	2	5	8	10	12
ΔV_{th} (V) - Device #6 With SVA	10.5	19.5	24	26	28.5
ΔI_{ph} (μA) - Device #5 W/O SVA	14.7	25.0	38.5	44.8	51.3
ΔI_{ph} (μA) - Device #6 With SVA	56.6	119.2	155.9	175.0	187.6
