# Supplementary material for: Diffusion interface layer controlling the acceptor phase of bilayer near-infrared polymer phototransistors with ultrahigh photosensitivity
Source: Nat Commun. 2022 Mar 11;13:1332. doi: 10.1038/s41467-022-28922-4 (PMC8917130; doi:10.1038/s41467-022-28922-4)
Supplement: Supplementary file 3 — Description of Additional Supplementary Information (Supplementary Movie 1) [file 41467_2022_28922_MOESM3_ESM.docx]

**Description of Additional Supplementary Information:**

**Supplementary Movie 1:** The video demonstrates the whole organic film fabrication process via film transfer method (FTM). The PDPP3T:PC_61_BM (0.2 wt%, donor:acceptor = 1:1) solution within 30 μL was dropped into a petri dish (diameter: 60 cm) filled with deionized water. When the CHCl_3_ solvent of PDPP3T:PC_61_BM solution volatilized completely, cutting and transferring the organic film by a tweezer to obtain the FTM-based film.
